# Endoscopic ultrasound-guided biliary drainage using a novel visibility enhancement mode of a fluoroscopic system

**DOI:** 10.1055/a-2418-0445

**Published:** 2024-10-02

**Authors:** Takeshi Ogura, Yuki Uba, Nobuhiro Hattori, Kimi Bessho, Hiroki Nishikawa

**Affiliations:** 1 38588 Endoscopy Center, Osaka Medical and Pharmaceutical University Hospital, Osaka, Japan; 2 38588 2nd Department of Internal Medicine, Osaka Medical and Pharmaceutical University Hospital, Osaka, Japan

Interventional endoscopic ultrasound (EUS) including EUS-guided biliary drainage (EUS-BD) has been indicated for failed endoscopic retrograde cholangiopancreatography (ERCP). The technical steps can be divided into four: bile duct puncture, guidewire insertion, tract dilation, and stent deployment 1 2 3 . During EUS-BD, a 0.025-inch guidewire is mainly used. In addition, fine-gauge devices such as a dilation device 4 or a stent delivery system 5 have been developed. However, these devices might provide poor visibility with contrast medium injection, especially EUS-guided hepaticogastrostomy (HGS). To improve the fluoroscopic visibility of these devices, a novel visibility enhancement mode of a fluoroscopic system (Astorex i9; Canon Medical Systems, Kanagawa, Japan), called Accent mode, has become available. Technical tips for EUS-HGS using Accent mode are presented.

A 77-year-old man was admitted to our hospital due to obstructive jaundice caused by cancer of the head of the pancreas. Biliary drainage was previously tried under ERCP guidance, but because of tumor invasion into the duodenum, EUS-HGS was attempted. The intrahepatic bile duct was punctured using a 19G needle, and the contrast medium was injected ( Fig. 1 ). Then, insertion of a 0.025-inch guidewire (VisiGlide; Olympus Medical, Tokyo, Japan) through the needle was attempted ( Fig. 2 ). However, on cholangiography, several bile duct branches were observed, and the visibility of the guidewire was inadequate. In this situation, if the guidewire were inserted into the bile duct branches, the visibility of the guidewire might be decreased. Therefore, we switched into Accent mode ( Fig. 3 ), thereby increasing the visibility of the guidewire. After successful guidewire deployment, a fine-gauge stent delivery system (5.9-Fr, Hanarostent Benefit; M.I. Tech, Seoul, S. Korea), whose visibility on fluoroscopic imaging was poor, was inserted. However, the distal end of the stent was clearly identified ( Fig. 4 ), and EUS-HGS was finally successful without any adverse events and the patient was discharged after 5 days ( Fig. 5 **,** Video 1 ).

**Fig. 1:**
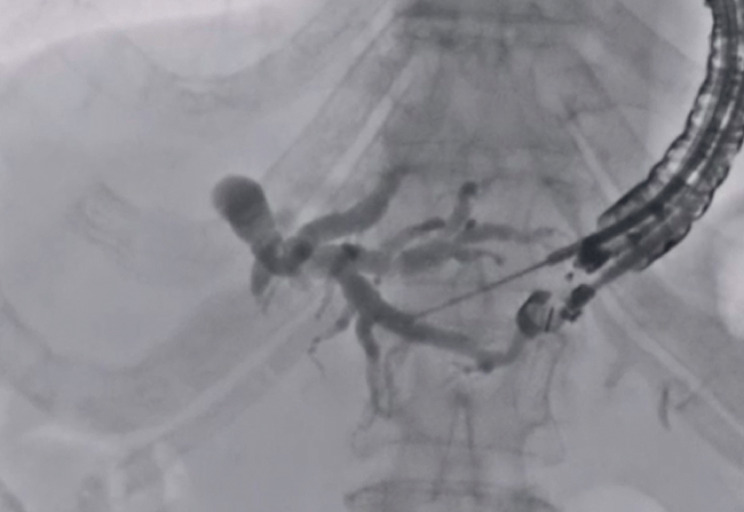
The intrahepatic bile duct is punctured using a 19G needle.

**Fig. 2:**
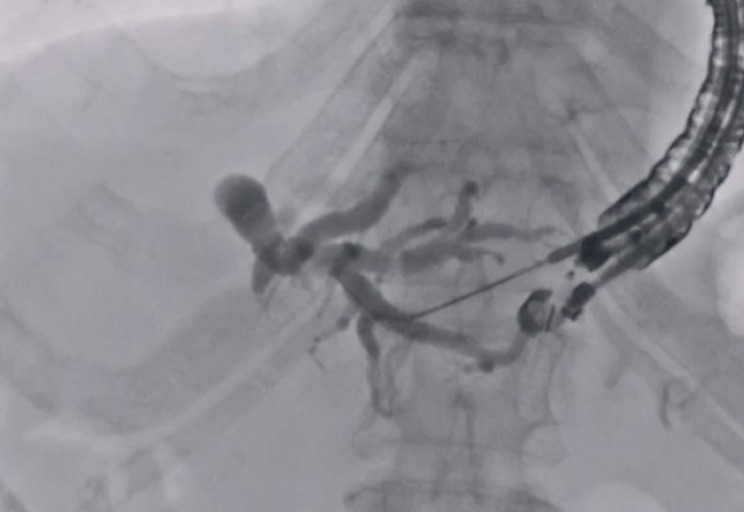
Insertion of a 0.025-inch guidewire is attempted, but the visibility of guidewire is inadequate.

**Fig. 3:**
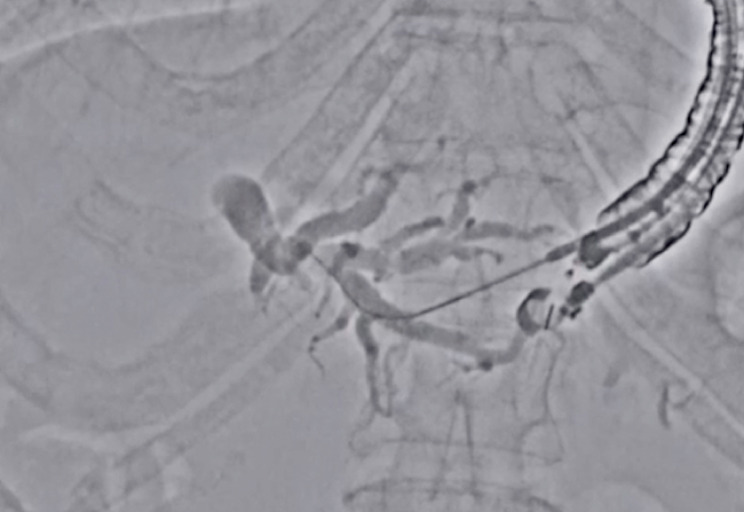
Accent mode improves the visibility of the guidewire.

**Fig. 4:**
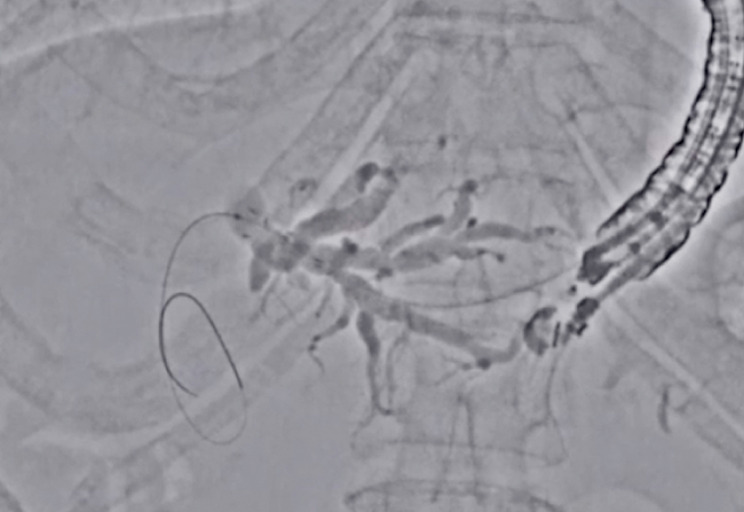
Stent release is attempted with adequate visibility.

**Fig. 5:**
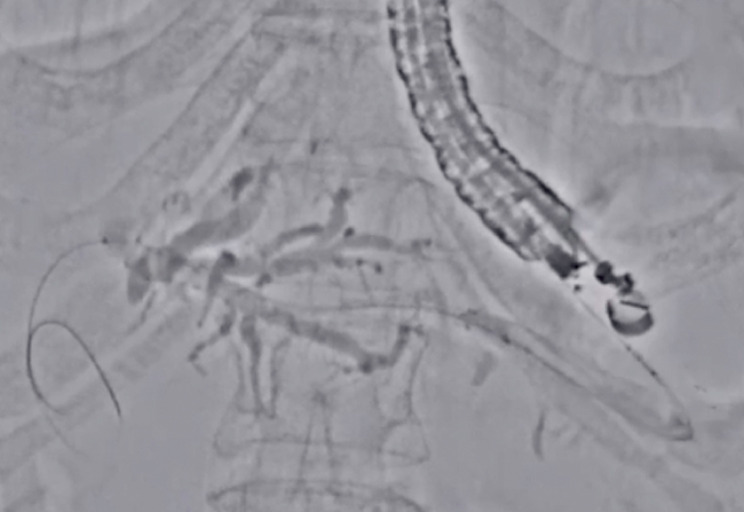
Stent deployment from the intrahepatic bile duct to the stomach is successfully performed.

Stent deployment is performed using Accent mode.Video 1

In conclusion, a novel visibility enhancement mode of a fluoroscopic system might be useful during EUS-BD, especially when using fine-gauge devices.

Endoscopy_UCTN_Code_TTT_1AS_2AH

## Notes

Endoscopy E-Videos https://eref.thieme.de/e-videos
*E-Videos* is an open access online section of the journal *Endoscopy* , reporting on interesting cases and new techniques in gastroenterological endoscopy. All papers include a high-quality video and are published with a Creative Commons CC-BY license. Endoscopy E-Videos qualify for HINARI discounts and waivers and eligibility is automatically checked during the submission process. We grant 100% waivers to articles whose corresponding authors are based in Group A countries and 50% waivers to those who are based in Group B countries as classified by Research4Life (see: https://www.research4life.org/access/eligibility/ ). This section has its own submission website at https://mc.manuscriptcentral.com/e-videos .
